# Senescent Endothelial Cells Sustain Their Senescence-Associated Secretory Phenotype (SASP) through Enhanced Fatty Acid Oxidation

**DOI:** 10.3390/antiox12111956

**Published:** 2023-11-02

**Authors:** Angelica Giuliani, Anna Maria Giudetti, Daniele Vergara, Laura Del Coco, Deborah Ramini, Sara Caccese, Matilde Sbriscia, Laura Graciotti, Gianluca Fulgenzi, Luca Tiano, Francesco Paolo Fanizzi, Fabiola Olivieri, Maria Rita Rippo, Jacopo Sabbatinelli

**Affiliations:** 1 Cardiac Rehabilitation Unit of Bari Institute, Istituti Clinici Scientifici Maugeri IRCCS, 70124 Bari, Italy; 2 Department of Biological and Environmental Sciences and Technologies, University of Salento, 73100 Lecce, Italy; anna.giudetti@unisalento.it (A.M.G.); daniele.vergara@unisalento.it (D.V.); laura.delcoco@unisalento.it (L.D.C.); fp.fanizzi@unisalento.it (F.P.F.); 3 Clinic of Laboratory and Precision Medicine, IRCCS INRCA, 60121 Ancona, Italy; d.ramini@inrca.it (D.R.); m.sbriscia@inrca.it (M.S.); f.olivieri@staff.univpm.it (F.O.); 4 Department of Clinical and Molecular Sciences, Università Politecnica delle Marche, 60126 Ancona, Italy; s.caccese@studenti.univpm.it (S.C.); g.fulgenzi@staff.univpm.it (G.F.); m.r.rippo@staff.univpm.it (M.R.R.); j.sabbatinelli@staff.univpm.it (J.S.); 5 Department of Biomedical Sciences and Public Health, Università Politecnica delle Marche, 60126 Ancona, Italy; l.graciotti@staff.univpm.it; 6 Department of Life and Environmental Sciences, Università Politecnica delle Marche, 60131 Ancona, Italy; l.tiano@staff.univpm.it; 7 Laboratory Medicine Unit, Azienda Ospedaliero Universitaria delle Marche, 60126 Ancona, Italy

**Keywords:** endothelial cells, senescence, fatty acid oxidation, senescence-associated secretory phenotype, glycolysis

## Abstract

Cellular senescence is closely linked to endothelial dysfunction, a key factor in age-related vascular diseases. Senescent endothelial cells exhibit a proinflammatory phenotype known as SASP, leading to chronic inflammation (inflammaging) and vascular impairments. Albeit in a state of permanent growth arrest, senescent cells paradoxically display a high metabolic activity. The relationship between metabolism and inflammation is complex and varies across cell types and senescence inductions. While some cell types shift towards glycolysis during senescence, others favor oxidative phosphorylation (OXPHOS). Despite the high availability of oxygen, quiescent endothelial cells (ECs) tend to rely on glycolysis for their bioenergetic needs. However, there are limited data on the metabolic behavior of senescent ECs. Here, we characterized the metabolic profiles of young and senescent human umbilical vein endothelial cells (HUVECs) to establish a possible link between the metabolic status and the proinflammatory phenotype of senescent ECs. Senescent ECs internalize a smaller amount of glucose, have a lower glycolytic rate, and produce/release less lactate than younger cells. On the other hand, an increased fatty acid oxidation activity was observed in senescent HUVECs, together with a greater intracellular content of ATP. Interestingly, blockade of glycolysis with 2-deoxy-D-glucose in young cells resulted in enhanced production of proinflammatory cytokines, while the inhibition of carnitine palmitoyltransferase 1 (CPT1), a key rate-limiting enzyme of fatty acid oxidation, ameliorated the SASP in senescent ECs. In summary, metabolic changes in senescent ECs are complex, and this research seeks to uncover potential strategies for modulating these metabolic pathways to influence the SASP.

## 1. Introduction

Cellular senescence plays a significant role in endothelial dysfunction, which in turn is a crucial aspect of age-related vascular diseases [1,2]. In fact, senescent endothelial cells acquire a characteristic proinflammatory secretory phenotype, i.e., SASP, which perpetuates the inflammatory state in the vascular environment [3], resulting in chronic low-grade inflammation, referred to as inflammaging [4]. This chronic inflammation disrupts the normal functioning of endothelial cells and therefore leads to impaired vasodilation, increased vascular permeability, and reduced anticoagulation [5,6]. Additionally, the inflammatory milieu attracts immune cells such as macrophages, which further amplify the proinflammatory response and contribute to tissue damage.

Despite the permanently arrested state of cell growth, senescent cells show high metabolic activity. Consequently, it appears that the inflammatory and metabolic conditions are intricately intertwined, with the former being fueled by the latter [7]. The first studies of the metabolic transition linked to the inflammatory condition were conducted on cells of innate immunity. Enhanced glycolysis was historically associated with inflammation and fatty acid oxidation (FAO) with anti-inflammation, but this vision may be an oversimplification [8]. Indeed, the metabolic shift during senescence appears to be cell-specific and dependent on the type of senescence induction [9]. For example, in vitro studies on the replicative senescence of mesenchymal stromal cells (MSC) evidenced that the metabolic shift from glycolysis to oxidative phosphorylation (OXPHOS) contributes to increased MSC heterogeneity, the abundance of senescent subsets, and altered secretory and migratory functions [10]. Consistently, human MSCs from old bone marrow donors showed a lower glucose uptake and glycolytic activity compared to those from younger donors, implying that these modifications are influenced not only by the replicative senescence resulting from repeated in vitro passages but also by chronological aging [11]. On the contrary, both DNA damage-induced and proliferative exhaustion-induced senescent human fibroblasts showed increased glycolysis compared with those of young proliferating cells [12].

Despite their large availability of oxygen, endothelial cells (ECs) opt for glycolysis rather than maximizing energy production through OXPHOS by diverting all the glucose to the surrounding tissues [13,14]. Historically, the metabolism of ECs has been investigated in mechanistic studies in the field of pathological angiogenesis. Data from senescence models are limited and display a high degree of heterogeneity depending on the senescence stimuli and tissue sources of ECs. Overall, while senescence is associated with enhanced glycolysis in most cell types [12,15], a relative impairment of glycolysis [16], accompanied by alterations in mitochondrial mass and dynamics [17], was consistently reported in ECs. In this framework, the connection between the metabolic reprogramming of ECs and the acquisition of a proinflammatory status that fuels age-related vascular dysfunction remains elusive.

Here, we aimed to characterize the basal metabolic profile of young and replicative senescent human umbilical vein endothelial cells (HUVECs) and subsequently explore the potential for modulating the overrepresented metabolic pathways in senescent ECs as a means to influence the SASP.

## 2. Materials and Methods

### 2.1. Cell Culture and Treatments

Cryopreserved human umbilical vein endothelial cells (HUVECs) pooled donors were purchased from Lonza (#CC-2519, Clonetics Lonza, Walkersville, MD, USA) and maintained in endothelial basal medium (EBM-2, #CC-3156, Clonetics Lonza) supplemented with EGM-2 SingleQuots (#CC-31622, Clonetics Lonza), with a final concentration of 2% of fetal bovine serum (FBS). Cells were maintained in incubator at 37 °C in a humidified atmosphere containing 5% CO_2_. HUVECs were seeded at a density of 5000 cells/cm^2^ and passaged when they achieved confluence until they reached a state of irreversible growth arrest. Population doublings (PDs) were calculated at every passage according to the following formula: log_10_F − log_10_I)/log_10_2, where F is the number of harvested cells and I the number of seeded cells. Cumulative PDs derive from the sum of PDs. For the 24-h treatment with L-carnitine, 2-deoxy-D-glucose, or etomoxir, cells were seeded on 6-well plates at a density of 8000 cells/cm^2^ and treated for 24 h.

### 2.2. Senescence-Associated Beta-Galactosidase

For detecting the senescence-associated beta-galactosidase (SA β-Gal) activity, the (BioVision Inc., Milpitas, CA, USA) kit was used. According to the producer’s suggested protocol, HUVECs cultured in a 24-well plate were fixed with the provided fixation solution. A quick room-temperature incubation (15–20 min) was followed by two PBS washes. Finally, cells were treated with an X-Gal solution and left incubating overnight at 37 °C. Acting as a β-Gal substrate, X-Gal was hydrolyzed and transformed into an insoluble blue pigment. Cells were considered “senescent” when the percentage of SA β-Gal was over 80%.

### 2.3. MTT Assay

MTT (3-(4,5-dimethylthiazol-2-yl)-2,5-diphenyltetrazolium bromide) assay was chosen for testing cellular viability. Mitochondrial enzymes catalyze the conversion of yellow tetrazolium salt into purple formazan compounds, detectable through spectrophotometric analysis. The assay was carried out under the same conditions for testing both 2-deoxy-D-glucose (2-DG), L-carnitine, and etomoxir. MTT solution (1 mg/mL) was added to HUVECs (grown in a 96-well plate) after a 24 h treatment and incubated for 4 h. The resulting formazan salts were solubilized with dimethyl sulfoxide (DMSO), and their absorbance at 540 nm was measured using a microplate reader (Biotek Synergy HTX, Agilent, Santa Clara, CA, USA). Untreated cells were considered as 100% vital.

### 2.4. Oxidative Damage Assay

The oxidative damage of proteins was determined by quantifying carbonyl groups by OxyBlotTM Protein Oxidation Detection Kit (Chemicon, Tokyo, Japan), according to the manufacturer’s recommendations. Briefly, carbonyl groups were derivatized with 2,4-dinitrophenylhydrazine to obtain 2,4-dinitrophenylhydrazone products. Non-derivatized samples were used as negative controls. Both derivatized and non-derivatized samples were separated by SDS-PAGE and transferred onto polyvinylidene difluoride membranes. Membranes were blocked with phosphate-buffered saline supplemented with 1% bovine serum albumin and 0.1% Tween 20 and incubated with a primary antibody (1:150) against DNP-modified carbonyl groups. Protein-antibody complexes were visualized using Amersham ECL Advance Western Blotting Detection Kit (Amersham, Piscataway, NJ, USA).

### 2.5. Immunoassays

IL-6 levels in the cellular medium were investigated using a commercial enzyme-linked immunosorbent assay (ELISA) kit (cat. no. 501030, Cayman, Ann Arbor, MI, USA). Supernatant cell cultures were collected and stored at −80 °C; after being centrifugated at 14,000 rpm for 20 min, the experiment was conducted in duplicate, following the kit procedure. The manufacturer assay range was 7.8–250 pg/mL. The expression of cytokines IL-1α, IL-1β, IL-6, IL-8, CXCL1 (Groα), CCL-2, CXCL10 (IP-10), and CCL5 (RANTES) was evaluated using Luminex reagents on a BioPlex^®®^ 200 analyzer (Bio-Rad, Hercules, CA, USA). The cell medium was collected and centrifugated at 1000× *g* for 15 min at 4 °C; the procedure followed was the producer’s recommended one. Briefly, a 96-well plate was filled with 50 µL standard, control, and samples. The various reagents (microparticle cocktail, diluted biotin-antibody cocktail, and diluted streptavidin-PE) were added, alternating with incubation and washing steps.

### 2.6. Nuclear Magnetic Resonance Analysis

Sample preparation was performed according to the procedure reported by Kostidis et al., 2017 [18]. Before analysis, CCM samples were centrifuged at 4 °C, and a volume of 500 µL was mixed with 100 µL of saline buffer solution at pH 7.4 (0.1 M K_2_HPO_4_ buffer in D_2_O containing trimethylsilyl propionic-2,2,3,3-*d*4 acid sodium salt, TSP) to minimize the pH variation and then transferred to a 5 mm NMR tube. The ^1^H NMR spectra (ZGCPPR experiments) were recorded at 300 K in automation mode using a Bruker Avance III NMR spectrometer (Bruker, Ettlingen, Germany) operating at 600.13 MHz for ^1^H observation, equipped with a TCI cryoprobe (Triple Resonance inverse Cryoprobe), and incorporating a *z*-axis gradient coil and automatic tuning-matching. For each sample, the following experimental conditions were used: 128 transients, 16 dummy scans, 5 s relaxation delay, FID (free induction decay) size of 64 K data points, spectral width of 12,019.230 Hz (20.0276 ppm), and acquisition time of 2.73 s. The resulting FIDs were multiplied by an exponential weighting function corresponding to a line broadening of 0.3 Hz before Fourier transformation, automated phasing, and baseline correction. Moreover, 2D NMR spectra (^1^H Jres, ^1^H-^1^H COSY, ^1^H-^13^C HSQC, and ^1^H-^13^C HMBC) were also randomly acquired for assignment purposes, together with a comparison with published data and public databases [18]. The NMR spectra were processed using Topspin 3.6.1 and Amix 3.9.13 (Bruker, Biospin, Milano, Italy), both for simultaneous visual inspection and the successive bucketing process for multivariate statistical analyses. The entire NMR spectra (in the range 9.5–0.5 ppm) were segmented in fixed rectangular buckets of 0.005 ppm width and successively integrated. The spectral regions between 5.23–4.68, 3.70–3.63, and 1.21–1.15 ppm, due to the residual peaks of solvents (water and ethanol), were discarded. The total sum normalization and the Pareto scaling procedure (performed by dividing the mean-centered data by the square root of the standard deviation) were applied to minimize minor differences due to sample concentration and/or experimental conditions among samples. Unsupervised principal component (PCA) and supervised partial least squares discriminant (PLS-DA) analyses were conducted in the webserver MetaboAnalyst 5.0 [19]. Variable importance in projection (VIP) scores of each variable in the PLS-DA model were analyzed, indicating the most discriminating metabolites in descending order of importance.

The box plots and corresponding one-way ANOVA following Tukey’s honest significant differences (HSD) post hoc test were calculated for discriminant metabolites among the compared groups. The level of statistical significance was considered as *p*-value < 0.05 with a 95% confidence interval.

### 2.7. Telomere Length

After genomic DNA isolation with QIAamp DNA Blood Kits (cat. no. 51104, Qiagen, Hilden, Germany), telomere length was analyzed by quantitative PCR using Cawthon’s method [20]. Briefly, the reference DNA sample was diluted serially in water by 1.68-fold dilution to produce DNA concentrations ranging from 6 to 0.23 ng in 5 µL. To reduce inter-assay variability, the telomere and the single-copy gene (36B4) were analyzed simultaneously. Primer sequences (written 5′→3′) were as follows:

tel 1, GGTTTTTGAGGGTGAGGGTGAGGGTGAGGGTGAGGGT;
tel 2, TCCCGACTATCCCTATCCCTATCCCTATCCCTATCCCTA;
36B4u, CAGCAAGTGGGAAGGTGTAATCC;
36B4d, CCCATTCTATCATCAACGGGTACAA.

The thermal cycling profile was as follows: (1) one cycle of 10 s at 95 °C and (2) 30 cycles of 5 s at 95 °C, 15 s at 57 °C, and 20 s at 72 °C. Measurements were performed in duplicate and reported as T/S ratio relative to a calibrator sample (Roche Applied Science, Indianapolis, IN, USA).

### 2.8. Total RNA Extraction

HUVECs’ RNA was isolated using the Norgen Biotek Kit (#37500, Norgen Biotek, Thorold, ON, Canada). The extraction procedure followed the manufacturer’s provided protocol. Until being analyzed, RNA was stored at −80 °C.

### 2.9. mRNA Expression

Purified RNA was spectrophotometrically quantified using Nanodrop ONE (NanoDrop Technologies, Wilmington, NC, USA). Reverse transcription of total RNA (1 ug) was conducted with TAKARA Kit (PrimeScript™ RT reagent Kit with gDNA Eraser, Cat: RR047A, Takara Bio, Shiga, Japan), following the producer’s recommended protocol. RT-PCR was run in Rotor Gene Q 5plex HRM (Qiagen, Germany), using TB Green Premix Ex Taq (Tli RNase H Plus) (RR420A, Takara Bio) in a 10 µL reaction volume, according to its indications of use. The mRNA gene expression was determined by the 2^−ΔCt^ method, with β-actin as a chosen control gene.

The primers sequences (written 5′–3′) were IL-1β Fw: TCTGCAGCTCTGTGTGTGAAGG, Rv: TGGGGTGGAAAGGTTTGGA; IL-6 Fw: CCAGCTACGAATCTCCGACC, Rv: CATGGCCACAACAATGACG; IL-8 Fw: TCTGCAGCTCTGTGTGTGAAGG, Rv: TGGGGTGGAAAGGTTTGGA; PAI-1 Fw: CAGATGAGGACAGAGTGGTTTC, Rv: GTGGGCTGATTAGGCTTGTATT; MPC1 Fw: CGGATGACATTTGCCCTCTG, Rv: GTGTTTGATAAGCCGCCCTC; LPL Fw: TGGAGGTACTTTTCAGCCAGGAT, Rv: TCGTGGGAGCACTTCACTAGCT; CD36 ATGTAACCCAGGACGCTGAG, Rv: GCCACAGCCAGATTGAGAAC, CPT1A Fw: CCACGATTCCACTCTGTCC, Rv: GCTGGATGGTGTCTGTCTCC, CPT1B Fw: CCAGGATCTGGGCTATGTGT, Rv: GGGCGCACAGACTCTAGGTA; FABP5 Fw: AGCTGGAAGGAAGATGGCG, Rv: TCCCACTCCTAGCTCCTTCA; PPARγ Fw: AGCCTCATGAAGAGCCTTCCA; Rv: ACCCTTGCATCCTTCACAAGC; ACOX1 Fw: CCCAGCTCATCACTAAGGGG, Rv: TTTGGGGCCGATGTCACC; ACAD Fw: CATGGAGCAACGTGTGTACC, Rv: TGAGGTCTTCGATCAGTGGG; CCL2 FW: AGCCACCTTCATTCCCCAAG; RV: TGGGACACTTGCTGCTGG; P16 Fw: CATAGATGCCGCGGAAGGT, Rv: CTAAGTTTCCCGAGGTTTCTCAGA; β-actin Fw: TGCTATCCCTGTACGCCTCT, Rv: GTGGTGGTGAAGCTGTAGCC.

### 2.10. Glucose and Lactate Measurement

Glucose and lactic acid in conditioned media were measured on an automated clinical chemistry analyzer (Atellica CH 930, Siemens Healthineers, Erlangen, Germany) using dedicated colorimetric methods.

### 2.11. ATP Assessment

For ATP quantification, CellTiter-Glo^®®^ Luminescent Cell Viability kit (Promega G7570, Madison, WI, USA) was used following the manufacturer’s instructions. Briefly, young and senescent HUVECs (10,000 cells per condition) were plated in a 96-well plate. After overnight attachment, cells were treated directly with CellTiter-Glo^®®^ Reagent. Mix contents for 2 min on an orbital shaker to induce cell lysis. Luminescence was recorded 10 min after reagent addition using a luminometer.

### 2.12. Fatty Acid Oxidation Assay and Extracellular O_2_ Consumption Assay

Fatty acid oxidation (FAO) was assessed in live HUVECs using the kit Fatty Acid Oxidation Assay purchased from Abcam (#ab217602, Cambridge, UK) in combination with another kit from Abcam, i.e., Extracellular Oxygen Consumption Assay (#ab197243), following the manufacturing’s instruction. Briefly, young and senescent HUVECs were seeded in a 96-well and allowed to attach overnight before the assay. Fluorescence was measured in a plate reader at 1.5 min intervals for 75 min at excitation/emission = 380/650 nm. The oxygen consumption rate (OCR) is presented as ΔFluorescence intensity/min/103 cells. At least three replicates were included for each measurement.

### 2.13. Western Blot Analysis

Proteins were extracted with RIPA lysis buffer (150 mM NaCl, 10 mM Tris, pH 7.2, 0.1% SDS, 1.0% Triton X-100, and 5 mM EDTA, pH 8.0), with a combination of protease and phosphatase inhibitors (Roche Applied Science, USA). After being measured with Bradford assay (Sigma-Aldrich, Milano, Italy), proteins (25 µg) were resolved through SDS-PAGE using Precast 4–15% Gel (Bio-Rad). The Trans-Blot Turbo™ Transfer system (Bio-Rad) ensured the transfer to a nitrocellulose membrane (Bio-Rad), which was blocked with 5% non-fat dried milk for 1 h at room temperature. Primary antibody was added and incubated overnight at 4 °C and subsequently probed with secondary horseradish peroxidase (HRP)-conjugated antibody (Vector, Oak Brook, IL, USA). Protein bands were visualized with ECL for detecting its chemiluminescent signal with UVITEC. Densitometric analysis was performed with ImageJ software (version 1.53, https://imagej.nih.gov/ij/download.html). Full and uncropped Western blots are provided as Supplementary Materials.

The primary antibodies used were anti-CPT1B (1:900, ab134988, Abcam, UK), FASN (1:1000 GTX109833, GeneTex, Irvine, CA, USA), p16/INK4a (1:200; sc-377,412, Santa Cruz Biotechnology, Dallas, TX, USA), LDH-A (1:1000, sc-137243, Santa Cruz Biotechnology, USA), HK-II (1:1000, sc-374091, Santa Cruz Biotechnology, USA), NF-κB p65 (1:1000 C22B4 #4764 Cell Signaling Technologies, Danvers, MA, USA), Phospho-NF-κB p65 (Ser536) (1:1000 93H1 #3033 Cell Signaling Technologies), and β-actin (1:5000 #3700 8H10D10 Cell Signaling Technologies). For phosphorylated protein, after stripping the membrane again, it was immunoblotted with anti-p65 (total NF-kB) antibody (Cell Signaling Technologies).

### 2.14. Transmission Electron Microscopy

For ultrastructural analyses, young and senescent HUVEC cells growing on Aclar films were fixed in 2.5% glutaraldehyde in 0.1 M cacodylate buffer (pH 7.4) and postfixed in osmium tetroxide 1% and then embedded in epoxy resin for TEM studies as previously described [17]. An MT-X ultratome (RMC; Tucson, AZ, USA) was used to obtain ultrathin sections (~50 nm). Ultrastructural characterization was performed on all samples using a CM10 Philips transmission electron microscope (FEI-Philips, Hillsboro, OR, USA).

### 2.15. Statistical Analysis

Data are presented as the mean ± standard deviation (SD) of at least three independent experiments. Student’s *t*-test was applied to determine differences between samples. One-way ANOVA was used to compare expression of three different conditions. Chi-square test was used to compare SA-β-gal data across conditions. *p*-values < 0.05 were considered significant. Statistical analyses were performed using GraphPad Prism v9.0 for windows (GraphPad Software, San Diego, CA, USA).

## 3. Results

### 3.1. Characterization of the Senescence Status of HUVECs

**Figure 1:**
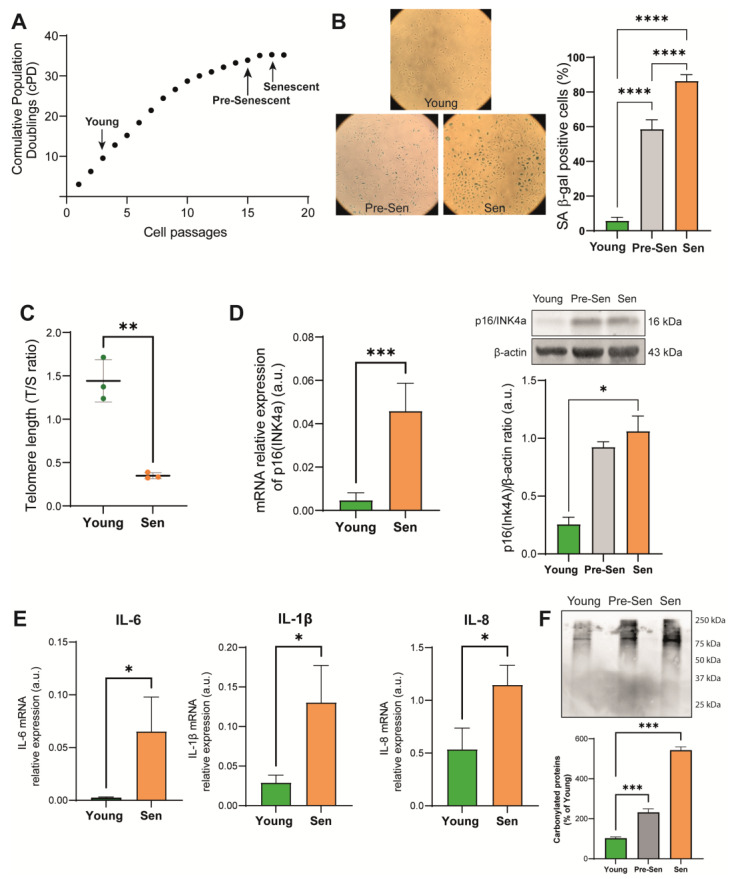
Characterization of replicative senescent in human umbilical vein endothelial cells (HUVEC). (**A**) HUVEC growth curve (cells passages, x axes; cumulative population doubling (cPD), y-axes); passages for young, pre-senescent, and senescent cells are highlighted. (**B**) Representative positivity of senescence-associated β-galactosidase images of young, pre-senescent (Pre-Sen), and senescent cells (Sen) and quantification. Magnification, 10×. (**C**) Telomere/single copy gene ratio (T/S) in DNA from young and senescent HUVECs. (**D**) p16/INK4a mRNA relative expression evaluated through real-time PCR reported as arbitrary units (a.u.) and Western blot and densitometric analysis of p16(INK4a) in young and SEN HUVECs. Protein expression values are reported as p16(INK4a)/β-actin ratio. (**E**) IL-6, IL-8, and IL-1β mRNA relative expression evaluated through real-time PCR. Β-actin was used as internal control. (**F**) Representative image of Oxyblot and its densitometric analysis; carbonylated proteins from young cells were taken as reference (100%). Data are represented as mean ± SD of three independent experiments. * *t*-test *p* < 0.05; ** *t*-test *p* < 0.01; *** *t*-test *p* < 0.001; **** *t*-test *p* < 0.0001.

To characterize basal metabolism of senescent ECs, HUVECs were cultured in vitro until they reached replicative arrest. EC senescence was documented by progressive reduction of population doubling (cPDs) in the cell growth curve (Figure 1A). Cells were considered senescent based on a >80% senescence-associated β-galactosidase (SA β-gal) positivity threshold (Figure 1B). To address the possibility that the replication arrest could significantly affect the metabolic profile of senescent ECs, we analyzed an additional group of cells harvested at two passages before occurrence of replication arrest (passage 15, Figure 1A), which we referred to as pre-senescent cells (Pre-Sen).

With continuous proliferation, the cells experience progressive telomere shortening until their exhaustion, leading to an irreversible mitotic arrest. Figure 1C shows the telomere shortening in senescent HUVECs evaluated using real-time PCR. Moreover, senescent ECs were characterized by significantly increased levels of the cell cycle regulator p16(INK4a) both at the transcriptional and protein level, as shown in Figure 1D, and of the proinflammatory SASP components interleukin (IL)-6, IL-1β, and IL-8 (Figure 1E). Replicative senescence was accompanied by progressive enhancement of protein oxidation assessed by the Oxyblot assay, which marks the carbonyl groups of oxidized proteins (Figure 1F).

### 3.2. Characteristics of the Basal Metabolism of Young and Senescent HUVECs

**Figure 2:**
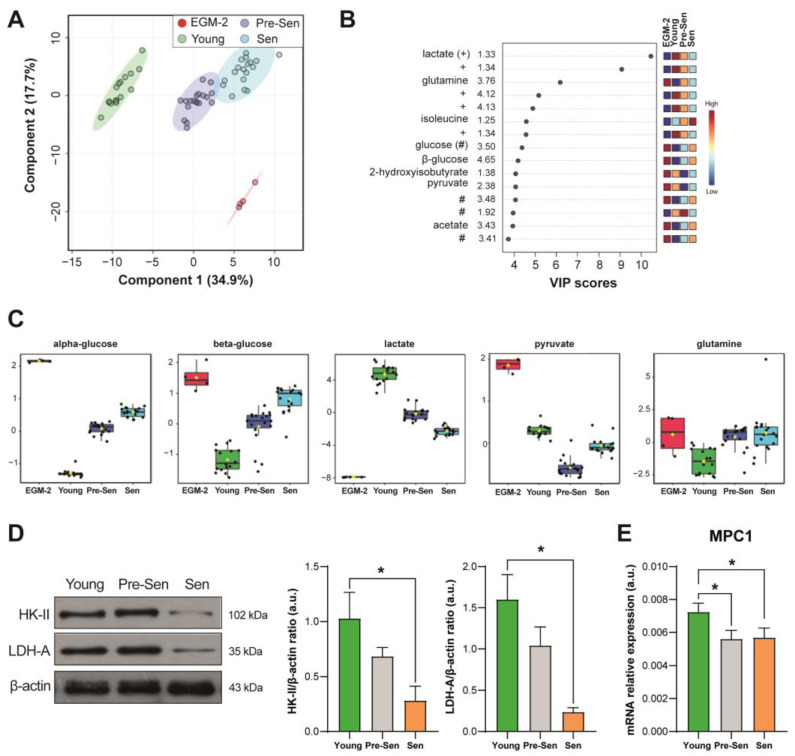
Metabolomic profiling of senescent HUVECs. (**A**) PLS-DA score plot distinguishing among plain culture medium (EGM-2) and young, pre-senescent, and senescent HUVECs. (**B**) Variable importance in projection (VIP) plot indicating the most discriminating buckets related to specific metabolites signals in descending order of importance. Multiple buckets identifying different other signals related to the same metabolite are also indicated for lactate (+) and glucose (#). Blue and red boxes denote metabolite levels that are lower or higher, respectively, between groups, with light orange and blue indicating intermediary levels. Only unbiased buckets related to ^1^H NMR metabolite signals are considered for further analyses, while bins referring to overlapping signals or to patterns of signals for the same molecule are reported. (**C**) Boxplots of selected metabolites differentially regulated among conditions (with relative *p*-values reported in Table S1). Y-axes are represented as relative units. Data were mean-centered and normalized to the total spectral area. Due to the mean-centering and normalization process, we obtained a negative scale in the y-axis of the bins. The bar plots show the normalized values (mean ± standard deviation). The boxes range from the 25% and the 75% percentiles; the 5% and 95% percentiles are indicated as error bars; single data points are indicated by black dots. (**D**) Western blot and densitometric analysis of hexokinase-II (HK-II) and lactate dehydrogenase A (LDHA). (**E**) mRNA relative expression. * *t*-test *p* < 0.05.

We performed a ^1^H nuclear magnetic resonance spectroscopy (NMR)-based metabolomic analysis of 72 h culture media of young and senescent endothelial cells. A representative ^1^H NMR spectrum is shown in Supplementary Figure S1A. The NMR spectra of culture media are dominated by several patterns of signals referred to as metabolites, such as amino acids, glucose, glycerol, lactate, pyruvate, and formate. After the NMR spectra preprocessing (phasing, baseline correction, and bucketing procedure; see Materials and Methods), multivariate data analysis was applied to the binned data. Both principal component analysis (PCA) (Supplementary Figure S1B) and partial least squares discriminant analysis (PLS-DA) (Figure 2A) demonstrate a clear separation of samples based on the different experimental conditions, with the greatest separation observed between young and senescent cells. In the PLS-DA model, the analysis of the fresh medium alone (EGM-2), containing several metabolites required for cell survival, is also reported for comparison. Only unbiased buckets related to ^1^H NMR metabolite signals are labeled in Figure 2B. These refer to metabolites in decreasing order of their ability to discriminate between the experimental conditions (VIP score), while the color code indicates the relative amount of each metabolite across conditions.

We performed a pathway enrichment analysis based on differentially regulated metabolites between senescent and young cells. The results of the analysis reported in Table 1 show that five metabolic pathways were enriched and differentially regulated in replicative senescent cells. Interestingly, the most enriched biochemical pathway was glycolysis (FDR = 1.78 × 10^−5^).

Based on NMR data, senescent HUVECs showed a lower glucose consumption compared to young HUVECs (Figure 2C). Accordingly, the expression of the glycolysis rate-limiting enzyme hexokinase II (HK-II) and the A subunit of lactate dehydrogenase (LDH-A), the enzyme that converts pyruvate into lactate, was reduced (Figure 2D). Moreover, NMR analysis showed a reduction in the levels of pyruvate that was not coupled with an increase in lactate in the conditioned media (Figure 2C). Furthermore, the mRNA level of the mitochondrial pyruvate carrier MPC-1, responsible for the import of pyruvate into the mitochondrion, was slightly reduced in senescent cells (Figure 2E). Pre-senescent and senescent ECs showed a significantly reduced utilization of glutamine compared to young cells (Figure 2C). The results of the metabolomic profiling suggested that metabolic reprogramming occurs in a gradual fashion with the onset of senescence and that replication arrest is not accompanied by abrupt changes in the metabolic phenotype of ECs. Thus, we performed the subsequent experiments considering only the extreme phenotypes of young and senescent HUVECs.

### 3.3. Lipid Metabolism in Senescent Endothelial Cells

**Figure 3:**
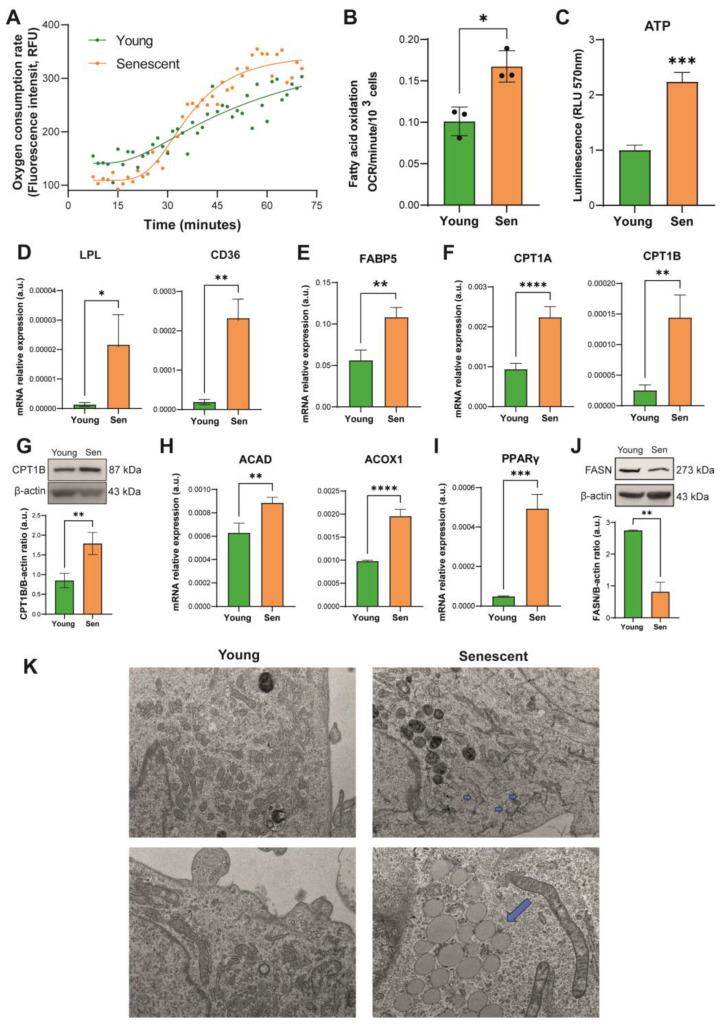
Lipid metabolism in senescent HUVECs. (**A**) Kinetic curve of beta-oxidation-related oxygen consumption rate (OCR) in young and senescent cells; (**B**) fatty acid oxidation, expressed as OCR/minute/10^3^ cells; (**C**) intracellular ATP content; (**D**) lipoprotein lipase (LPL) and CD36 mRNA relative expression. (**E**) Fatty-acid-binding protein 5 (FABP5) mRNA expression. (**F**) Carnitine palmitoyl transferase 1 (CPT1) A and B mRNA relative expression. (**G**) Western blot and densitometric analysis of CPT1B. Protein expression values are reported as CPT1B/β-actin ratio. (**H**) Acyl-CoA dehydrogenase (ACAD) and peroxisomal acyl-coenzyme A oxidase 1 (ACOX1) mRNA relative expression. (**I**) PPAR-γ mRNA relative expression. (**J**) Western blot and densitometric analysis of fatty acid synthase (FASN) in young and senescent HUVECs. Protein expression values are reported as FASN/β-actin ratio. (**K**) Representative TEM image of young and senescent HUVECs, original magnification 7.5 K (upper panels) and 19 K (lower panels). Arrows are pointing lipid droplet most frequently found in senescent cells (upper right image). In some senescent cells, the lipids droplets were represented well in the cell cytoplasm (bottom right image). Data are represented as mean ± SD of three independent experiments. * *t*-test *p* < 0.05; ** *t*-test *p* < 0.01; *** *t*-test *p* < 0.001; **** *t*-test *p* < 0.0001.

Having verified that senescent endothelial cells do not internalize and consume large amounts of glucose from the culture medium, we went on to analyze fatty acid metabolism. For this reason, we first assayed oxygen consumption and fatty acid oxidation (FAO) of senescent HUVECs compared to their younger counterpart. These assays measure the oxygen consumption rate (OCR) following the addition of oleic acid to a medium depleted of fatty acids. Before the assay, cells were treated overnight with a glucose-deprived medium. Treatment with etomoxir, an inhibitor of medium-chain fatty acid mitochondrial import, was used as a negative control. A kinetic curve (Figure 3A) lasting 75 min was constructed, and the OCR was evaluated as the variation of fluorescence intensity per hour between minutes 5–70 and normalized according to the number of seeded cells, which was equal across conditions. A significant increase in the FAO rate by senescent HUVECs was reported (Figure 3B), which can be also appreciated by the visual inspection of the kinetic curves. Therefore, we sought to analyze the bioenergetic impact of enhanced expression of FAO on senescent HUVECs. The concentration of ATP, which reflects the global efficiency of the bioenergetic pathways, was determined in the cell lysate and normalized to the number of cultured cells. We observed a significant increase in intracellular ATP levels in senescent cells (Figure 3C).

To deeply investigate lipid metabolism, we analyzed the mRNA expression of several selected genes. RT-PCR analysis showed an increased transcription of lipoprotein lipase (LPL) and CD36, used by endothelial cells for the extraction of triacylglycerols from lipoproteins and the internalization of long-chain fatty acids, respectively (Figure 3D). Accordingly, an increase in fatty-acid-binding protein 5 (FABP5), which binds cytosolic fatty acids and thus facilitates fatty uptake in ECs (Figure 3E) and carnitine palmitoyl transferase 1A and 1B (CPT1A and CPT1B), which participate in the mitochondrial translocation of fatty acids and represent rate-limiting enzymes for beta-oxidation, was observed in senescent HUVECs (Figure 3F,G). Furthermore, the acyl-CoA dehydrogenase (ACAD) and acyl-CoA oxidase 1 (ACOX1), which catalyze the first reaction of beta-oxidation in mitochondria and peroxisomes, respectively, were significantly upregulated (Figure 3H). Accordingly, we found that peroxisome proliferator-activated receptor gamma (PPARγ), a transcriptional factor regulating genes involved in lipid metabolism, is upregulated in senescent HUVECs (Figure 3I). On the other hand, the protein expression of fatty acid synthase (FASN), which catalyzes endogenous fatty acid biogenesis, was reduced in senescent HUVECs (Figure 3J). Finally, transmission electron microscope (TEM) analysis revealed the presence of lipid droplets in the cytoplasm of senescent ECs, which likely reflects the accumulation of exogenous triacylglycerols by HUVECs (Figure 3K).

### 3.4. Blockade of Glycolysis Increases the Expression of Proinflammatory Cytokines in Endothelial Cells

Since senescent HUVECs overexpress a plethora of proinflammatory molecules characterizing the SASP phenotype, we sought to verify whether this might be attributable to alterations in their metabolism.

**Figure 4:**
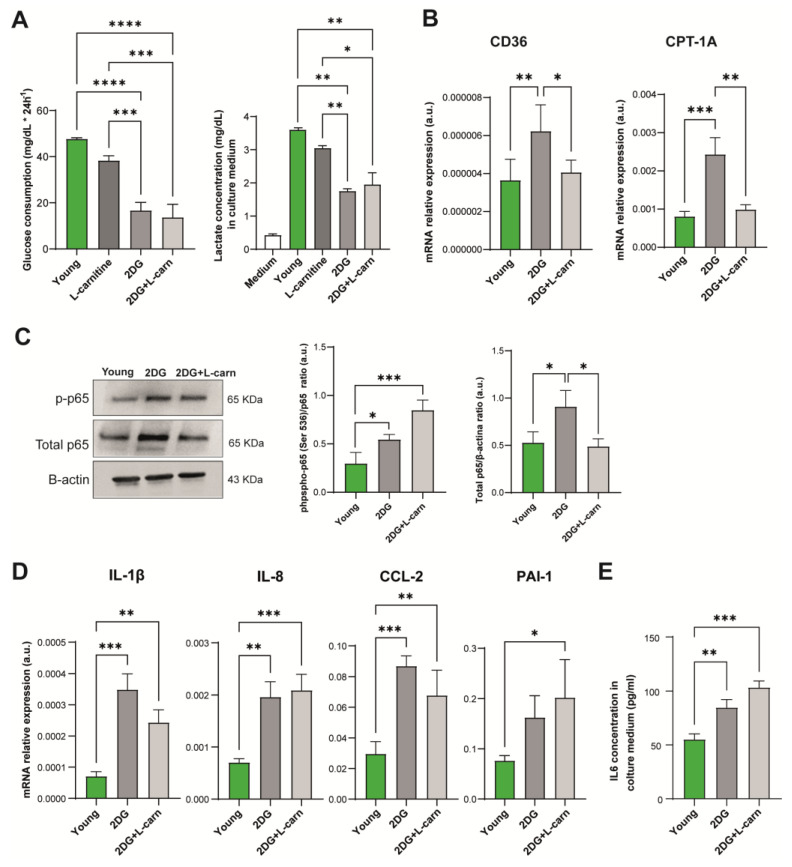
(**A**) Glucose consumption and lactate concentration in culture medium in young HUVECs treated for 24 h with 2-deoxyglucose (2DG), L-carnitine, or both. (**B**) CD36 and CPT1A mRNA relative expression in young cells. (**C**) Protein expression of phosphorylated and total p65(NF-kB) and relative densitometric analysis. The amount of p-65(NF-kB) is expressed as ratio to total p65. (**D**) IL-1beta, IL-8, CCL-2, and PAI-1 mRNA expression. (**E**) Concentration of IL-6 released into the culture medium after 24 h of treatment expressed as pg/mL. * *t*-test *p* < 0.05; ** *t*-test *p* < 0.01; *** *t*-test *p* < 0.001; **** *t*-test *p* < 0.0001.

For this reason, we analyzed the effects of perturbations of the metabolic status of endothelial cells, treating young HUVECs with a combination of 2-deoxyglucose (2-DG) and L-carnitine for 24 h to inhibit glycolysis. Optimal 2-DG and L-carnitine treatment concentrations were determined as 0.1 mM and 2.5 mM for 2-DG and L-carnitine, respectively, based on MTT results (Supplementary Figure S2A). Glycolysis inhibition was analyzed by measuring glucose consumption and lactate production by ECs in a conditioned medium (Figure 4A). 2-DG and L-carnitine were also tested in combination, yielding a similar viability on HUVECs. As L-carnitine alone did not induce any significant modulation of the glycolytic rate (Figure 4A), we used only 2-DG and 2-DG+L-carnitine for further experiments. 2-DG treatment induced expression of CD36, suggesting an increase in the import of fatty acids from outside the cell, and CPT1A (Figure 4B), the enzyme catalyzing the rate-limiting step of FAO. On the contrary, ACAD mRNA expression was not influenced by the treatment (Supplementary Figure S2C).

Treatment with 2-DG alone induced transcription and phosphorylation of NF-kB p65 subunit (Figure 4C), while 2-DG in association with L-carnitine induced p65 phosphorylation (Figure 4C). Accordingly, enhanced transcription of the downstream IL-1β, IL-8, and CCL-2 (Figure 4D) and an increased release of IL-6 into the medium (Figure 4E) was observed in young HUVECs treated with 2-DG alone or combined with L-carnitine, while an increase in PAI-1 expression occurred only after treatment with 2DG+L-carnitine (Figure 4D). Interestingly, the short-term treatment with 2-DG and L-carnitine, while affecting the expression of a number of SASP mediators, did not induce a significant modulation of the senescent state, assessed in terms of p16 expression (Supplementary Figure S2D).

### 3.5. Blockade of Fatty Acid Oxidation Reduces the Expression of Proinflammatory Cytokines in Senescent Endothelial Cells

**Figure 5:**
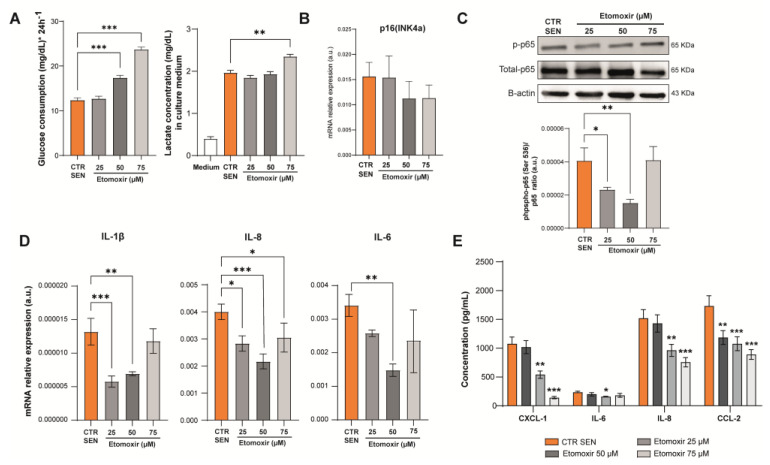
(**A**) Glucose consumption and lactate production of senescent HUVECs treated with the CPT1 inhibitor etomoxir at different concentrations (25 µM, 50 µM, and 75 µM) for 24 h. (**B**) mRNA expression of p16(INK4a). (**C**) Protein expression of phosphorylated and total p65(NF-kB) and relative densitometric analysis. The amount of p-65(NF-kB) is expressed as ratio to total p65. (**D**) mRNA expression of IL-1β, IL-8, and IL-6. (**E**) Pro-inflammatory cytokine (CXCL-1, IL-6, IL-8, and CCL-2) concentration in culture medium of SEN cells treated with etomoxir. * *t*-test *p* < 0.05; ** *t*-test *p* < 0.01; *** *t*-test *p* < 0.001.

As senescent cells use fatty acids as a preferential source for energy production, to evaluate a possible association between their metabolic phenotype and the proinflammatory phenotype, we blocked FAO in senescent HUVECs with etomoxir, an inhibitor of CPT1, which is a key protein involved in the transport of fatty acids into mitochondria. First, the toxicity of etomoxir on senescent cells was evaluated by MTT. Based on the results, we decided to perform experiments with three different concentrations (25 µM, 50 µM, and 75 µM) (Supplementary Figure S2B). Senescent HUVECs treated with 50 and 75 µM etomoxir displayed increased glucose consumption, which was accompanied by increased lactate release into the medium only with 75 µM etomoxir (Figure 5A).

After observing that p16 mRNA levels were not affected by 24 h etomoxir treatment (Figure 5B), we evaluated the expression of the SASP components. The activation of NF-kB in senescent cells, assessed in terms of phosphorylation of its p65 subunit, was significantly rescued by 24 h treatment with 25 or 50 µM etomoxir (Figure 5C). The transcription of IL-1β, IL-8, and IL-6 mRNAs was down-regulated in senescent cells treated with 50 µM etomoxir compared to control (untreated senescent cells) (Figure 5D).

To better characterize the effects of metabolism modulation on the SASP, we measured the levels of various proinflammatory cytokines released into the culture medium using a multiplexed immunoassay. The data confirmed that treatment with etomoxir reduces the release of the proinflammatory cytokines CXCL-1, IL-6, IL-8, and CCL-2 in the conditioned medium (Figure 5E).

## 4. Discussion

Numerous studies suggest that senescent cells undergo a profound remodeling of metabolic pathways [21,22,23]. Even if the general picture appears very complex, since the specific metabolic alterations are different depending on the cell types and pro-senescence stimuli, a general trend towards a more glycolytic state in senescent cells can be observed in a broad range of cell types, including ECs [24,25].

The aim of this study was to evaluate the metabolic reprogramming of endothelial cells in replicative senescence and a potential link with their proinflammatory status and maintenance of the SASP. Endothelial cells represent a good model for the study of replicative senescence and inflammation [6]; moreover, endothelial dysfunction is involved in the pathogenesis and progression of numerous age-associated diseases, such as type II diabetes and atherosclerosis [26].

In HUVECs, the cellular model investigated by our study, we confirmed that proliferating endothelial cells use glucose as their main energy source and demonstrated that a metabolic switch towards FAO occurs during replicative senescence. Our data on endothelial cells, which add to the growing body of evidence from other cellular senescence models [27], i.e., mostly dermal fibroblasts, should be interpreted in the light of the peculiar model employed. Indeed, ECs display a strong glycolytic phenotype, which protects them from the accumulation of ROS and preserves oxygen for the needs of perivascular cells [14]. Hence, a perturbation of this metabolic status could be expected during replicative senescence. Whether enhanced FAO could occur because of the senescent status or represent itself a driver of senescence-associated endothelial dysfunction remains to be clarified. Interestingly, an increased rate of FAO was observed also in models of senescence induced by stimuli other than replication arrest, such as oncogene-induced senescence (OIS) [27,28].

Our metabolic analysis highlighted a greater consumption of glucose and an increased release of lactic acid by the young cells, which together with a greater expression of lactate dehydrogenase (LDH-A), supports an increased activity of anaerobic glycolysis in physiological conditions. The data are also confirmed by the significant enrichment pathways analysis of metabolites belonging to the glycolytic pathway. On the contrary, the glycolytic activity seems to be reduced in senescent cells, in which, in fact, a reduced expression of HK-II, one of the rate-limiting enzymes of glycolysis, has been observed.

The significant increase in intracellular ATP levels in senescent HUVECs, in the presence of limited glycolytic activity, led us to hypothesize that endothelial cells in replicative senescence may rely on FAO for their bioenergetic needs. To confirm this hypothesis, we performed a functional assay evaluating the FAO rate on viable cells, which demonstrated that senescent cells uptake and metabolize exogenous fatty acids at a faster pace. Indeed, by acquiring electron microscopy images, we observed that senescent cells tend to accumulate triacylglycerols in the form of lipid droplets. These results are in line with other studies that observed that exogenous lipids are preferentially incorporated into triacylglycerols, which form the lipid droplets in senescent cells [29,30].

This observation, together with the increased expression of LPL and of the scavenger receptor CD36, supports the hypothesis that senescent endothelial cells have a marked tendency to capture fatty acids to be used for bioenergetic purposes. Interestingly, increased expression of CD36 has been observed in a variety of cell types in response to replicative, oncogenic, and chemical pro-senescence stimuli [31]. Moreover, Chong and colleagues demonstrated that ectopic CD36 expression triggers the activation of NF-kB, coupled with the production of SASP mediators including IL-6 and IL-8 [31]. More recently, it has been demonstrated that CD36 also regulates SASP in vivo [32]. In this context, it has been demonstrated that PPARγ is crucial for the CD36-mediated fatty acid uptake in ECs [33]. Interestingly, we observed an upregulation of PPARγ in senescent ECs.

As further confirmation of the increased oxidation of fatty acids, we observed an increased expression of the enzymes ACAD and ACOX1, which catalyze the first step of beta-oxidation at the mitochondrial and peroxisomal level, respectively, and of the CPT1 component of the carnitine-fatty acid shuttle, which is responsible for importing the latter into the mitochondrion. Together with the observation of reduced expression of the de novo lipogenesis enzyme FASN, we can assume that senescent ECs internalize and accumulate exogenous fatty acids rather than synthesizing them. The activation of FASN was proven to be required for the induction of the senescent phenotype and to sustain senescent cell metabolism in hepatic stellate cells [34]. On the contrary, oncogene-induced senescence of human fibroblasts was associated with increased FAO and reduced lipid biosynthesis [27]. Besides the specific cell lineage and senescence stimulus, the distinctive metabolic characteristics of ECs should be considered in the interpretation of our findings. First, de novo lipogenesis is required to maintain EC homeostasis by promoting eNOS palmitoylation and bioavailability [35]. Moreover, FASN deletion was shown to reduce EC proliferation and angiogenesis [36]. Notably, our findings agree with the concomitant observation of accelerated senescence, enhanced FAO, and reduced lipid biosynthesis in a model of coronary microvascular ECs treated with miR-30 mimics [37].

To demonstrate that the observed bioenergetic alterations are functional to the acquisition of the proinflammatory phenotype typical of cellular senescence, we used the compound etomoxir, which, by blocking the CPT1 activity, prevents the mitochondrial import of medium-chain fatty acids [38]. Treatment with etomoxir proved to be effective in negatively modulating the expression of the proinflammatory cytokines IL-6, IL-8, and IL-β and the release of CXCL-1, IL-6, IL-8, and CCL-2 in the conditioned medium, data supported by the lower expression of the active form of the p65 subunit of NF-κB. Of note, FAO blockade did not impact of p16 expression between untreated and treated senescent HUVECs. We believe that a short-term (24 h) treatment with etomoxir could be not sufficient to ameliorate p16 expression and thus revert the replication arrest observed in senescent HUVECs. Moreover, it has to be noted that etomoxir at high concentration (>100 µM) exerts a number of off-target effects that are independent from CPT1 blockade, such as mitochondrial complex I [39] and adenine nucleotide translocase (ANT) inhibition [40]. Therefore, we cannot exclude that our results could be related also to the inhibition of the oxidative phosphorylation, which is accountable for the higher ATP content observed in senescent HUVECs. Moreover, although etomoxir concentrations comparable to those used in the present study were reported to induce oxidative stress in T lymphocytes [41], here, we showed that its administration to senescent HUVECs ameliorated the SASP without significantly affecting cell viability. A further limitation that needs to be addressed regards the use of the MTT assay to assess cell viability for compounds affecting the metabolic phenotype of recipient cells. Indeed, reduction in the dye used for the assay depends primarily on cell metabolism, thus potentially leading to the inaccurate utility of the assay [42]. However, a previous study showed that MTT yielded similar results compared to the manual count in cells treated with up to 200 µM etomoxir for 48 h [39]. Regarding treatment with 2-DG, the use of MTT assay was extensively reported in the literature for concentrations comparable to those of our experiments [43,44,45].

## 5. Conclusions

Overall, our data suggest that in HUVEC, replicative senescence is associated with a metabolic reprogramming towards fatty acid oxidation. As a result, senescent ECs display a higher ATP content, which is functional to sustain the high-energy-demanding SASP. In this framework, the blockade of FAO can mitigate the SASP. Albeit limited to a single cellular lineage, our findings add to the body of evidence describing heterogeneous metabolic alterations in senescent cells. Understanding the mechanisms underlying endothelial dysfunction, inflammation, and metabolic alterations in senescent endothelial cells is crucial for developing therapeutic strategies to mitigate many age-related vascular diseases, including type 2 diabetes and atherosclerosis [46]. With this study, the hypothesis that switching off inflammation by targeting the metabolic pathways may be a potential therapeutic approach to improve endothelial function and preventing age-related vascular disorders can be suggested. Further studies additionally on animal and disease models are necessary to develop metabolism-directed interventions that can preserve endothelial function and prevent age-related vascular diseases.

## Figures and Tables

**Table 1:** Results of the pathway enrichment analysis. Pathways significantly enriched in metabolites differentially expressed among conditions are reported.

Metabolic Pathways	Total	Expected	Hits	Raw p	−Log_10_(p)	FDR
Glycolysis/gluconeogenesis	26	0.17	5	2.11 × 10^−7^	6.67	1.78 × 10^−5^
Glyoxylate and dicarboxylate metabolism	32	0.21	4	2.89 × 10^−5^	4.54	1.21 × 10^−3^
Aminoacyl tRNA biosynthesis	48	0.31	4	1.49 × 10^−4^	3.83	4.16 × 10^−3^
Pyruvate metabolism	22	0.14	3	2.80 × 10^−4^	3.55	5.87 × 10^−3^
Valine, leucine, and isoleucine biosynthesis	8	0.05	2	1.03 × 10^−3^	2.99	1.73 × 10^−2^

Total, the total number of compounds involved in the pathway; Hits, the number effectively matched by the data uploaded by the user; Raw p, the original *p*-value calculated from the enrichment analysis; FDR p, the *p*-value rectified using the false-discovery rate.

## Data Availability

The data presented in this study are available on request from the corresponding author.

## Supplementary Materials

The following supporting information can be downloaded at: https://www.mdpi.com/article/10.3390/antiox12111956/s1, Figure S1: (A) Typical 1H NMR spectrum of CCM (ZGCPPR experiment) and metabolite assignments. (B) Principal component analysis (PCA) performed for the NMR data; Figure S2: Results of the MTT assay performed to assess (A) viability and FAO rate of senescent HUVECs treated with etomoxir and (B) young HUVECs treated with 0.05–2.5 mM 2-DG and 0.05–10 mM L-carnitine for 24 h. (C–D) ACAD and p16(INK4a) mRNA expression in young HUVECs treated with 2-DG alone or in combination with L-carnitine; Table S1: ANOVA post hoc test for discriminant metabolites. Tukey honest significant difference (HSD) test for multiple comparisons of groups was also applied (*p*-value < 0.05).

## References

[1] Wiley C.D., Campisi J. (2021). The metabolic roots of senescence: Mechanisms and opportunities for intervention. Nat. Metab..

[2] Sabbatinelli J., Ramini D., Giuliani A., Recchioni R., Spazzafumo L., Olivieri F. (2021). Connecting vascular aging and frailty in Alzheimer’s disease. Mech. Ageing Dev..

[3] Coppé J.P., Patil C.K., Rodier F., Sun Y., Munoz D.P., Goldstein J., Nelson P.S., Desprez P.Y., Campisi J. (2008). Senescence-associated secretory phenotypes reveal cell-nonautonomous functions of oncogenic RAS and the p53 tumor suppressor. PLoS Biol..

[4] Franceschi C., Bonafe M., Valensin S., Olivieri F., De Luca M., Ottaviani E., De Benedictis G. (2000). Inflamm-aging. An evolutionary perspective on immunosenescence. Ann. N. Y. Acad. Sci..

[5] Ajoolabady A., Pratico D., Vinciguerra M., Lip G.Y.H., Franceschi C., Ren J. (2023). Inflammaging: Mechanisms and role in the cardiac and vasculature. Trends Endocrinol. Metab..

[6] Han Y., Kim S.Y. (2023). Endothelial senescence in vascular diseases: Current understanding and future opportunities in senotherapeutics. Exp. Mol. Med..

[7] Sabbatinelli J., Prattichizzo F., Olivieri F., Procopio A.D., Rippo M.R., Giuliani A. (2019). Where Metabolism Meets Senescence: Focus on Endothelial Cells. Front. Physiol..

[8] Van den Bossche J., O’Neill L.A., Menon D. (2017). Macrophage Immunometabolism: Where Are We (Going)?. Trends Immunol..

[9] Callender L.A., Carroll E.C., Bober E.A., Henson S.M. (2018). Divergent mechanisms of metabolic dysfunction drive fibroblast and T-cell senescence. Ageing Res. Rev..

[10] Yuan X., Logan T.M., Ma T. (2019). Metabolism in Human Mesenchymal Stromal Cells: A Missing Link Between hMSC Biomanufacturing and Therapy?. Front. Immunol..

[11] Barilani M., Lovejoy C., Piras R., Abramov A.Y., Lazzari L., Angelova P.R. (2022). Age-related changes in the energy of human mesenchymal stem cells. J. Cell. Physiol..

[12] James E.L., Michalek R.D., Pitiyage G.N., de Castro A.M., Vignola K.S., Jones J., Mohney R.P., Karoly E.D., Prime S.S., Parkinson E.K. (2015). Senescent human fibroblasts show increased glycolysis and redox homeostasis with extracellular metabolomes that overlap with those of irreparable DNA damage, aging, and disease. J. Proteome Res..

[13] Eelen G., de Zeeuw P., Treps L., Harjes U., Wong B.W., Carmeliet P. (2018). Endothelial Cell Metabolism. Physiol. Rev..

[14] Li X., Sun X., Carmeliet P. (2019). Hallmarks of Endothelial Cell Metabolism in Health and Disease. Cell. Metab..

[15] Zwerschke W., Mazurek S., Stockl P., Hutter E., Eigenbrodt E., Jansen-Durr P. (2003). Metabolic analysis of senescent human fibroblasts reveals a role for AMP in cellular senescence. Biochem. J..

[16] Kuosmanen S.M., Sihvola V., Kansanen E., Kaikkonen M.U., Levonen A.L. (2018). MicroRNAs mediate the senescence-associated decline of NRF2 in endothelial cells. Redox Biol..

[17] Giuliani A., Cirilli I., Prattichizzo F., Mensa E., Fulgenzi G., Sabbatinelli J., Graciotti L., Olivieri F., Procopio A.D., Tiano L. (2018). The mitomiR/Bcl-2 axis affects mitochondrial function and autophagic vacuole formation in senescent endothelial cells. Aging (Albany NY).

[18] Kostidis S., Addie R.D., Morreau H., Mayboroda O.A., Giera M. (2017). Quantitative NMR analysis of intra- and extracellular metabolism of mammalian cells: A tutorial. Anal. Chim. Acta.

[19] Xia J., Psychogios N., Young N., Wishart D.S. (2009). MetaboAnalyst: A web server for metabolomic data analysis and interpretation. Nucleic Acids Res..

[20] Cawthon R.M. (2002). Telomere measurement by quantitative PCR. Nucleic Acids Res..

[21] Aird K.M., Zhang R. (2014). Metabolic alterations accompanying oncogene-induced senescence. Mol. Cell. Oncol..

[22] Nakao M., Tanaka H., Koga T. (2020). Cellular Senescence Variation by Metabolic and Epigenomic Remodeling. Trends Cell Biol..

[23] Gitenay D., Wiel C., Lallet-Daher H., Vindrieux D., Aubert S., Payen L., Simonnet H., Bernard D. (2014). Glucose metabolism and hexosamine pathway regulate oncogene-induced senescence. Cell. Death Dis..

[24] Stabenow L.K., Zibrova D., Ender C., Helbing D.L., Spengler K., Marx C., Wang Z.Q., Heller R. (2022). Oxidative Glucose Metabolism Promotes Senescence in Vascular Endothelial Cells. Cells.

[25] Wiley C.D., Campisi J. (2016). From Ancient Pathways to Aging Cells-Connecting Metabolism and Cellular Senescence. Cell. Metab..

[26] Donato A.J., Machin D.R., Lesniewski L.A. (2018). Mechanisms of Dysfunction in the Aging Vasculature and Role in Age-Related Disease. Circ. Res..

[27] Quijano C., Cao L., Fergusson M.M., Romero H., Liu J., Gutkind S., Rovira I.I., Mohney R.P., Karoly E.D., Finkel T. (2012). Oncogene-induced senescence results in marked metabolic and bioenergetic alterations. Cell. Cycle.

[28] Kaplon J., Zheng L., Meissl K., Chaneton B., Selivanov V.A., Mackay G., van der Burg S.H., Verdegaal E.M., Cascante M., Shlomi T. (2013). A key role for mitochondrial gatekeeper pyruvate dehydrogenase in oncogene-induced senescence. Nature.

[29] Maeda M., Scaglia N., Igal R.A. (2009). Regulation of fatty acid synthesis and Delta9-desaturation in senescence of human fibroblasts. Life Sci..

[30] Flor A.C., Wolfgeher D., Wu D., Kron S.J. (2017). A signature of enhanced lipid metabolism, lipid peroxidation and aldehyde stress in therapy-induced senescence. Cell. Death Discov..

[31] Chong M., Yin T., Chen R., Xiang H., Yuan L., Ding Y., Pan C.C., Tang Z., Alexander P.B., Li Q.J. (2018). CD36 initiates the secretory phenotype during the establishment of cellular senescence. EMBO Rep..

[32] Moiseeva V., Cisneros A., Sica V., Deryagin O., Lai Y., Jung S., Andres E., An J., Segales J., Ortet L. (2023). Author Correction: Senescence atlas reveals an aged-like inflamed niche that blunts muscle regeneration. Nature.

[33] Abumrad N.A., Cabodevilla A.G., Samovski D., Pietka T., Basu D., Goldberg I.J. (2021). Endothelial Cell Receptors in Tissue Lipid Uptake and Metabolism. Circ. Res..

[34] Fafian-Labora J., Carpintero-Fernandez P., Jordan S.J.D., Shikh-Bahaei T., Abdullah S.M., Mahenthiran M., Rodriguez-Navarro J.A., Niklison-Chirou M.V., O’Loghlen A. (2019). FASN activity is important for the initial stages of the induction of senescence. Cell. Death Dis..

[35] Wei X., Schneider J.G., Shenouda S.M., Lee A., Towler D.A., Chakravarthy M.V., Vita J.A., Semenkovich C.F. (2011). De novo lipogenesis maintains vascular homeostasis through endothelial nitric-oxide synthase (eNOS) palmitoylation. J. Biol. Chem..

[36] Bruning U., Morales-Rodriguez F., Kalucka J., Goveia J., Taverna F., Queiroz K.C.S., Dubois C., Cantelmo A.R., Chen R., Loroch S. (2018). Impairment of Angiogenesis by Fatty Acid Synthase Inhibition Involves mTOR Malonylation. Cell. Metab..

[37] Veitch S., Njock M.S., Chandy M., Siraj M.A., Chi L., Mak H., Yu K., Rathnakumar K., Perez-Romero C.A., Chen Z. (2022). MiR-30 promotes fatty acid beta-oxidation and endothelial cell dysfunction and is a circulating biomarker of coronary microvascular dysfunction in pre-clinical models of diabetes. Cardiovasc. Diabetol..

[38] Divakaruni A.S., Hsieh W.Y., Minarrieta L., Duong T.N., Kim K.K.O., Desousa B.R., Andreyev A.Y., Bowman C.E., Caradonna K., Dranka B.P. (2018). Etomoxir Inhibits Macrophage Polarization by Disrupting CoA Homeostasis. Cell Metab..

[39] Yao C.H., Liu G.Y., Wang R., Moon S.H., Gross R.W., Patti G.J. (2018). Identifying off-target effects of etomoxir reveals that carnitine palmitoyltransferase I is essential for cancer cell proliferation independent of beta-oxidation. PLoS Biol..

[40] Kalucka J., Bierhansl L., Conchinha N.V., Missiaen R., Elia I., Bruning U., Scheinok S., Treps L., Cantelmo A.R., Dubois C. (2018). Quiescent Endothelial Cells Upregulate Fatty Acid beta-Oxidation for Vasculoprotection via Redox Homeostasis. Cell Metab..

[41] O’Connor R.S., Guo L., Ghassemi S., Snyder N.W., Worth A.J., Weng L., Kam Y., Philipson B., Trefely S., Nunez-Cruz S. (2018). The CPT1a inhibitor, etomoxir induces severe oxidative stress at commonly used concentrations. Sci. Rep..

[42] Ghasemi M., Turnbull T., Sebastian S., Kempson I. (2021). The MTT Assay: Utility, Limitations, Pitfalls, and Interpretation in Bulk and Single-Cell Analysis. Int. J. Mol. Sci..

[43] Liu H., Jiang C.C., Lavis C.J., Croft A., Dong L., Tseng H.Y., Yang F., Tay K.H., Hersey P., Zhang X.D. (2009). 2-Deoxy-D-glucose enhances TRAIL-induced apoptosis in human melanoma cells through XBP-1-mediated up-regulation of TRAIL-R2. Mol. Cancer.

[44] Pruss M., Dwucet A., Tanriover M., Hlavac M., Kast R.E., Debatin K.M., Wirtz C.R., Halatsch M.E., Siegelin M.D., Westhoff M.A. (2020). Dual metabolic reprogramming by ONC201/TIC10 and 2-Deoxyglucose induces energy depletion and synergistic anti-cancer activity in glioblastoma. Br. J. Cancer.

[45] Vijayan V., Pradhan P., Braud L., Fuchs H.R., Gueler F., Motterlini R., Foresti R., Immenschuh S. (2019). Human and murine macrophages exhibit differential metabolic responses to lipopolysaccharide—A divergent role for glycolysis. Redox Biol..

[46] Eelen G., de Zeeuw P., Simons M., Carmeliet P. (2015). Endothelial cell metabolism in normal and diseased vasculature. Circ. Res..

