# Supplementary material for: Senescent Endothelial Cells Sustain Their Senescence-Associated Secretory Phenotype (SASP) through Enhanced Fatty Acid Oxidation
Source: Antioxidants (Basel). 2023 Nov 2;12(11):1956. doi: 10.3390/antiox12111956 (PMC10668971; doi:10.3390/antiox12111956)
Supplement: Supplementary file 1 [file antioxidants-12-01956-s001.zip › antioxidants-2653175-supplementary.pdf]

Figure S1. (A) Typical 1H NMR spectrum of CCM (ZGCPPR experiment) and metabolite assignments. (B) Principal Componenent analysis (PCA) performed for the NMR data.

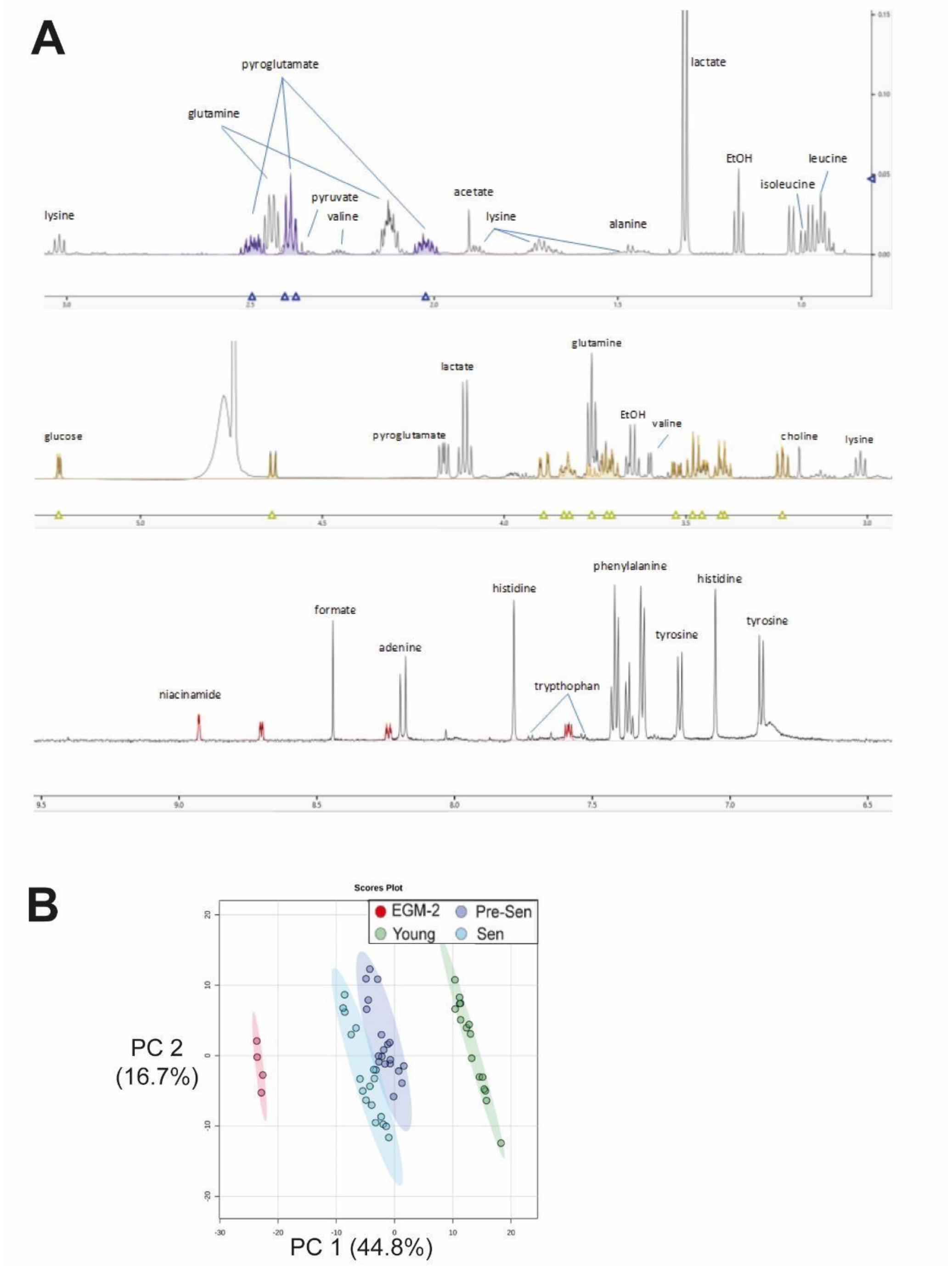

Figure S2. Results of the MTT assay performed to assess (A) viability and FAO rate of senescent HUVECs treated with etomoxir and (B) young HUVECs treated with 0.05-2.5 mM 2-DG and 0.05-10 mM L-carnitine for 24h. (C-D) ACAD and p16(INK4a) mRNA expression in young HUVECs treated with 2DG alone or in combination with L-carnitine.

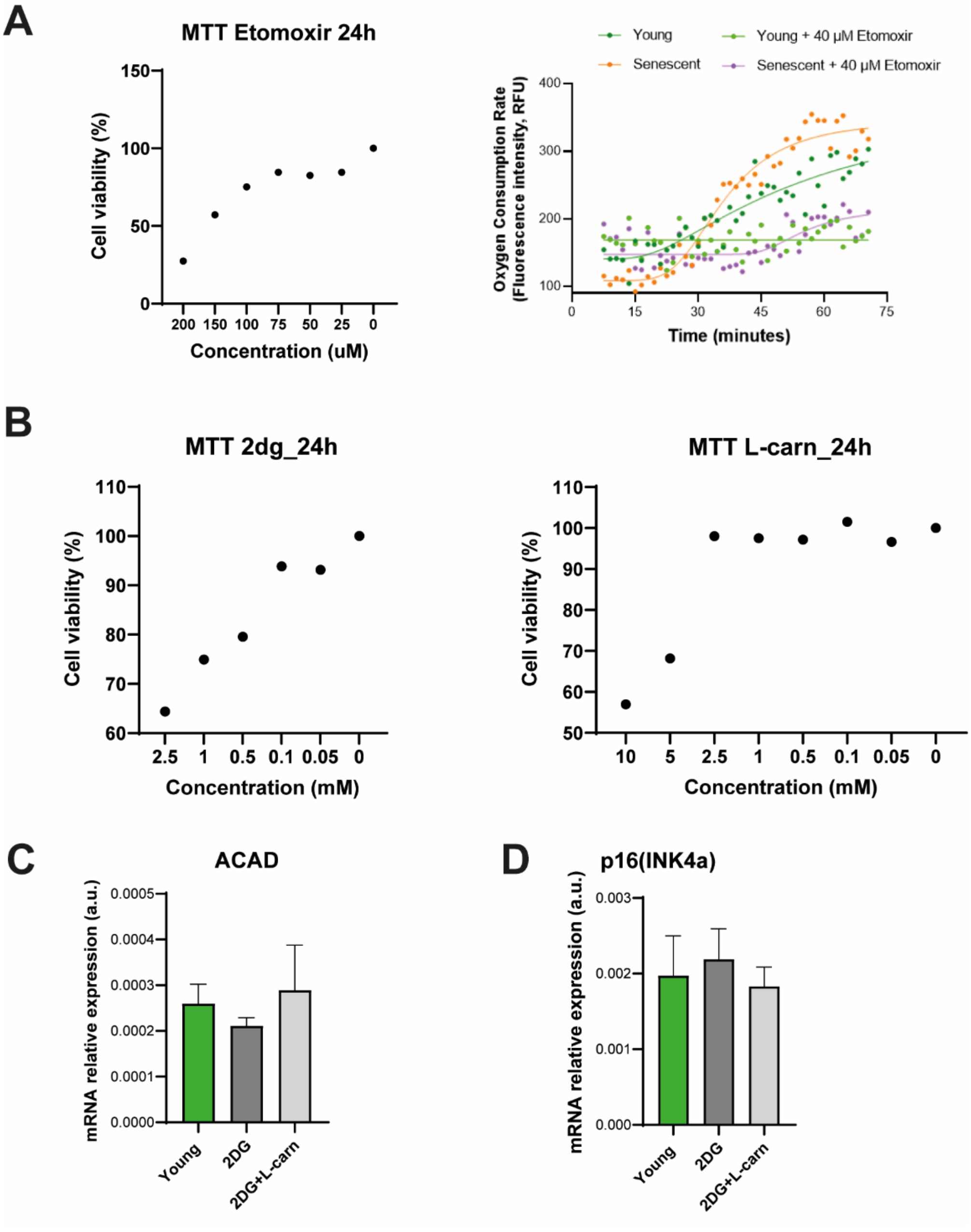

Table S1. Anova post-hoc test for discriminant metabolites. Tukey Honestly Significant Difference (HSD) test for multiple comparisons of groups was also applied (p-value <0.05).

Metabolite	NMR variable (ppm)	p-value	Tukey's HSD
alpha-glucose	5.24	$2.36*10^{-45}$	Pre-sen-EGM-2; Sen-EGM-2; Young-EGM-2; Sen-Pre-sen; Young-Pre-sen; Young-Sen
beta-glucose	4.64	$4.58*10^{-17}$	Pre-sen-EGM-2; Young-EGM-2; Sen-Pre-sen; Young-Pre-sen; Young-Sen
glutamine	3.76	$1.70*10^{-5}$	Young-EGM-2; Young-Pre-sen; Young-Sen
lactate	1.32	$4.37*10^{-12}$	Pre-sen-EGM-2; Sen-EGM-2; Young-EGM-2; Sen-Pre-sen; Young-Pre-sen; Young-Sen
pyruvate	2.38	$1.09*10^{-29}$	Pre-sen-EGM-2; Sen-EGM-2; Young-EGM-2; Sen-Pre-sen; Young-Pre-sen; Young-Sen
